# Case Report: Next-Generation Sequencing Identified a Novel Pair of Compound-Heterozygous Mutations of *LPL* Gene Causing Lipoprotein Lipase Deficiency

**DOI:** 10.3389/fgene.2022.831133

**Published:** 2022-03-03

**Authors:** Yakun Li, Man Hu, Lin Han, Lifang Feng, Luhong Yang, Xiaoqian Chen, Tingting Du, Hui Yao, Xiaohong Chen

**Affiliations:** ^1^ Department of Endocrinology and Metabolism, Wuhan Children’s Hospital, Tongji Medical College, Huazhong University of Science and Technology, Wuhan, China; ^2^ Running Gene Inc., Beijing, China

**Keywords:** LPL gene, lipoprotein lipase deficiency (LPLD), familial hyperchylomicronemia syndrome (FCS), hyperlipoproteinemia, whole-exome sequencing (WES)

## Abstract

Lipoprotein lipase deficiency (LPLD) is a rare disease characterized by the accumulation of chylomicronemia with early-onset. Common symptoms are abdominal pain, hepatosplenomegaly, eruptive xanthomas and lipemia retinalis. Serious complications include acute pancreatitis. Gene *LPL* is one of causative factors of LPLD. Here, we report our experience on an asymptomatic 3.5-month-old Chinese girl with only milky blood. Whole-exome sequencing was performed and identified a pair of compound-heterozygous mutations in *LPL* gene, c.862G>A (p.A288T) and c.461A>G (p.H154R). Both variants are predicted “deleterious” and classified as “likely pathogenic”. This study expanded the *LPL* mutation spectrum of disease LPLD, thereby offering exhaustive and valuable experience on early diagnosis and proper medication of LPLD.

## 1 Introduction

Lipoprotein lipase deficiency (LPLD, OMIM #238600), also called familial hyperchylomicronemia syndrome (FCS) or type 1 hyperlipoproteinemia, is characterized by uncontrolled accumulation of chylomicronemia (Chait and Eckel, 2019). The frequency of LPLD is approximately 1 per million in the most populations (Brahm and Hegele, 2015), except that it is much more common in the province of Quebec, Canada due to founder effect (Gagne et al., 1989). This condition is a rare hereditary disorder with which patients usually developed signs or symptoms before the age of 10 (early-onset), with 25% presenting symptoms by the first year of life (Burnett et al., 1993). Clinical manifestations of LPLD include recurrent abdominal pain, acute pancreatitis, hepatosplenomegaly, eruptive xanthomas and lipemia retinalis. Acute pancreatitis is the most serious consequence of severe hypertriglyceridemia, with a mortality rate of 2–5% (Lowenfels et al., 2009; Omdal et al., 2011). Neurological features such as depression and intellectual decline have also been reported in patients with LPLD. Fortunately, these symptoms can be remedied after blood lipid levels normalize.

In LPLD patients, lipoprotein lipase (LPL) is usually malfunctional or absent. The measurement of LPL enzyme activity in an assay system is an approach to establish the diagnosis but it is not routinely available. Since homozygous or a pair of compound-heterozygous variants in lipoprotein lipase protein gene (*LPL*, OMIM #609708) is the causative factor, the current mainstream approach to diagnosing LPLD is to detect biallelic pathogenic variants in *LPL* gene. According to Human Gene Mutation Database (HGMD) professional version (Stenson et al., 2017), a total of 214 disease-causing mutations (DM) in *LPL* have been reported to be associated with LPLD or relevant phenotypes. Analysis of the distribution of mutations and expanding the mutation spectrum are necessary for further diagnosis and research on LPLD. Herein, we report the clinical, biochemical and genetic findings of a 3.5-month-old Chinese girl diagnosed with LPLD, who carries a pair of compound-heterozygous *LPL* variants including a novel one. We expanded the spectrum of *LPL* mutations associated with LPLD. We also summarized, illustrated and analyzed mutations reported worldwide.

## 2 Methods

This research was approved by the Ethics Committee of Wuhan Children’s Hospital (No.2017020). The patient’s guardians were informed with a written consent for the investigation and publication of this study.

### 2.1 Clinical Data Collection

Clinical information of the patient was collected from official medical records and follow-up visits. Physical and biochemical examinations were performed in each visit, including lipid blood test, complete blood count (CBC), blood gas analysis, blood ammonia assay, blood lactic acid, blood sugar, coagulogram, antibody testing, urine organic acids (OAU) test. Abdominal and pelvic CT scanning, chest and abdominal X-ray imaging, brain MRI, brain magnetic resonance angiography (MRA), abdominal color doppler flow imaging (CDFI) and electroencephalogram (EEG) were performed. Blood amino acid and acylcarnitine profile was also analyzed by tandem mass spectrometry.

### 2.2 Whole-Exome Sequencing

Peripheral blood of the patient and her parents were collected and sent to Running Gene Inc. (Beijing, China) for whole-exome sequencing (WES). Genomic DNA samples were extracted using Blood DNA Kit V2 (#CW2553, Cowin Bio., Taizhou, China). Qubit dsDNA HS Assay Kit (#Q32851, Invitrogen, Carlsbad, CA) was used to determine concentrations. Gel electrophoresis were also performed for quality control. Qualified DNA samples were fragmented into 200–300 bp by sonication and then processed for DNA libraries preparation according to the manufacturer’s protocol of KAPA LTP Library Preparation Kit (#KR0453, Kapa Biosystems, Wilmington, MA), which includes four standard steps: end-repair, A-tailing, adapter ligation and library amplification. Cleanups during each steps were carried out with Agencourt AMPure XP reagent (#A63882, Beckman Coulter, Brea, CA). Evaluation of prepared libraries were performed using Qubit dsDNA HS Assay Kit. Capture probes hybridized to pooled DNA libraries using the Agilent SureSelectXT2 Target Enrichment System (Agilent, Santa Clara, CA). Target fragments (exome) were fished out by hybridization with Dynabeads^®^ MyOne™ Streptavidin T1 (#65601, Invitrogen). Purifications were performed using Agencourt AMPure XP reagent. Final libraries were evaluated by Qubit dsDNA HS Assay Kit. DNA libraries were sequenced on the Illumina NovaSeq 6000 platform as paired-end 150-bp reads.

Raw data of WES were stored as FASTQ format and processed by Fastp (Chen et al., 2018) for quality control. Qualified reads were aligned to human genome reference sequence (GRCh37/hg19) using Burrows-Wheeler Alignment (BWA) (Li and Durbin, 2009). Consensus single nucleotide polymorphisms (SNPs) and insertions and deletions (indels) were called by Genome Analysis Toolkit (GATK) (Van der Auwera et al., 2013). After quality control, called variants were annotated based on several public databases, such as 1kGenome (Genomes Project et al., 2015), ExAC (Lek et al., 2016), gnomAD (Karczewski et al., 2020), ESP6500 (Fu et al., 2013), ClinVar (Landrum et al., 2020), HGMD and in-house databases. Annotated variants were then filtered based on their relationship with disease and pathogenicity. The pathogenicity of candidate variants were predicted by multiple *in silico* algorithms, including MutationTaster2 (Schwarz et al., 2014), SIFT (Sim et al., 2012), Provean (Choi and Chan, 2015), Polyphen-2 (HDIV and HVAR) (Adzhubei et al., 2013), LRT (Chun and Fay, 2009), FATHMM (Shihab et al., 2013) and Mutpred2 (Pejaver et al., 2020). Variants were also classified based on American College of Medical Genetics and Genomics (ACMG) guidelines (Richards et al., 2015). Candidate variants, also including mutations in *PLIN1* gene (familial partial lipodystrophy type IV) and *SAR1B* gene (chylomicron retention disease), were then sent to Sanger for validation. Likely pathogenic variants were finally selected on the basis of their relationship to the disease, allele frequency in controls, pattern of segregation with disease, pattern of inheritance and predicted pathogenicity.

## 3 Results

### 3.1 Clinical Presentation

A 3.5-month-old Chinese girl admitted to our hospital due to her pneumonia with fever and vomiting, but we found her blood with milky appearance during blood routine examination. She is the second child of her non-consanguineous parents (G2P2). She was born by caesarean section in the 40th gestational weeks, with a birth weight of 3.8 kg (90 percentile) and a birth height of 50 cm (50 percentile). She was under breast-fed and her mom’s blood lipids were in the normal range (Supplementary Table 1). No abnormalities were found during pregnancy and delivery. No relevant medical history or family history was reported.

### 3.2 Physical Examinations

Her weight was 6 kg (25 percentile) and height was 63 cm (50 percentile) at the age of 3.5 months. A hemangioma-like red rash (1.0 × 1.0 cm) were identified on her right hypochondrium. The rash was excluded from eruptive xanthomas by dermatologists. Her liver is present 1.5 cm below the costal margin. No abnormalities found on her facial appearance, psychomotor development or muscular tension. No other relevant signs or symptoms were reported.

### 3.3 Laboratorial Examinations

Lipid blood test revealed elevated blood triglycerides (TG) and cholesterols (CHOL) levels as well as decreased levels of low-density lipoproteins (LDL), high-density lipoproteins (HDL), apolipoprotein A1 (ApoA1) and apolipoprotein B (ApoB) (Table 1). Normal results were shown by other biochemical examinations mentioned above.

**TABLE 1:** Biochemical examinations of blood lipid and lipoprotein levels.

Patient age	CHOL (mmol/L)		TG (mmol/L)		HDL (mmol/L)		LDL (mmol/L)		ApoA1 (g/L)		ApoB (g/L)	
3m16d	12.58	↑	67.84	↑	0.73	↓	0.24	↓	0.7	↓	0.27	↓
3m19d	5.79	↑	15.61	↑	0.35	↓	1.86	-	0.91	↓	1.86	↑
3m23d	6.74	↑	10.11	↑	0.27	↓	4.26	↑	NA		NA	
4m21d	3.89	-	9.41	↑	0.25	↓	< 0.315	↓	NA		NA	
Discharged												
6m14d	11.31	↑	74.10	↑	0.22	↓	0.11	↓	0.82	↓	0.38	↓
6m17d	6.09	↑	20.75	↑	0.34	↓	0.57	↓	0.74	↓	0.78	-
8m	6.14	↑	7.28	↑	0.30	↓	0.42	↓	0.88	↓	0.97	-
11m	6.10	↑	19.14	↑	0.44	↓	1.27	↓	0.94	↓	1.05	-
1y4m	4.20	-	3.94	↑	0.61	↓	2.69	-	0.86	↓	0.98	-
1y7m	4.73	-	2.20	↑	0.60	↓	3.05	↑	0.89	↓	1.49	↑
1y10m	4.50	-	3.53	↑	0.65	↑	3.07	↑	0.87	↓	1.20	-
2y1m	4.69	-	1.49	↑	0.83	↑	3.41	↑	1.20	-	1.09	-
2y5m	3.50	-	0.66	-	1.63	-	1.72	-	1.59	-	0.48	-
Reference	2.8–5.7		0.32–1.46		0.9–1.74		1.55–2.86		1.1–1.95		0.45–1.4	

ApoA1, apolipoprotein A-1; ApoB, apolipoprotein B; CHOL, cholesterol; HDL, high density lipoprotein; LDL, low density lipoprotein; LP (a), lipoprotein (a); TG, triglyceride; TG: 1 mol/L = 88.5 mg/dl.

Abdominal and pelvic CT scanning with and without contrast presented peripancreatic effusion which indicates the occurrence of pancreatitis. Chest and abdominal X-ray imaging showed increased lung texture. Blood amino acid and acylcarnitine profile analysis revealed decreased levels of multiple amino acids (methionine, leucine, valine, glycine, glutamine and threonine), which may be caused by malnutrition. No clinically significant abnormalities were identified by brain MRI, MRA, abdominal CDFI and EEG.

### 3.4 Genetic Analysis

**FIGURE 1:**
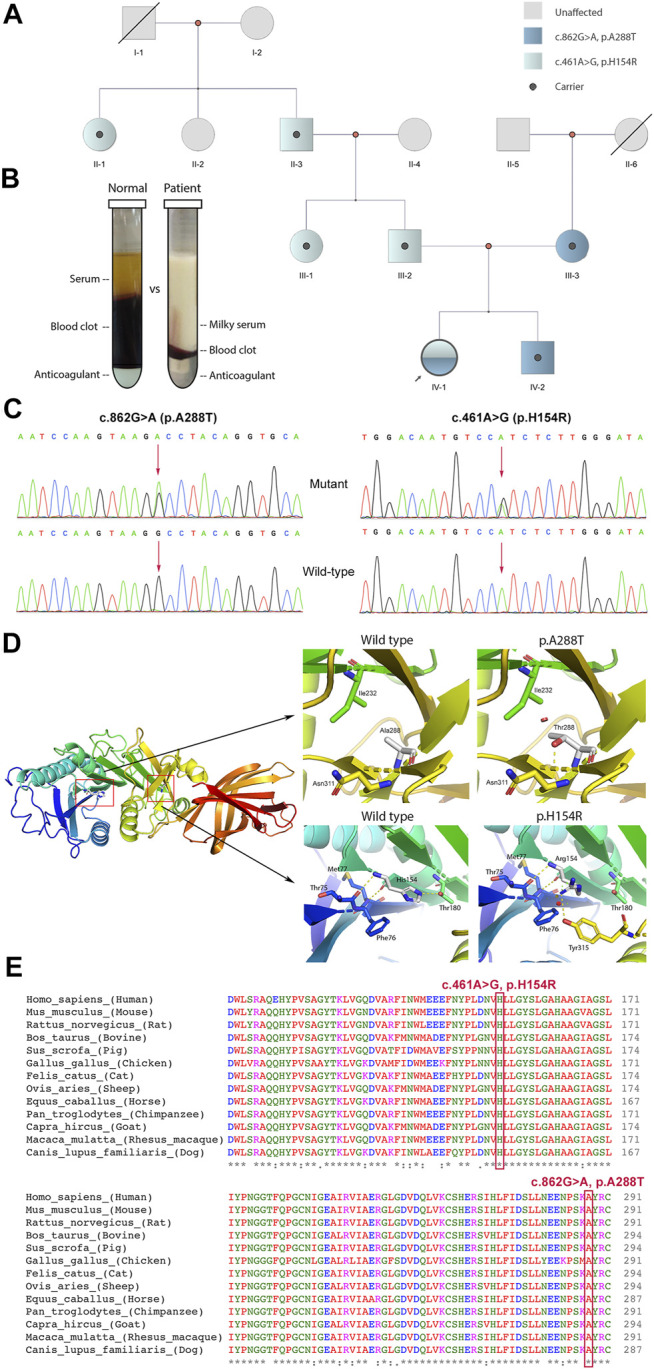
Clinical and genetic information of the patient. **(A)** Pedigree analysis of this family. **(B)** Blood fractionations (set for 24 h) between control and the patient. **(C)** Sanger sequencing of variants c.862G>A and c.461A>G with corresponding wild-types. **(D)** Three-dimensional protein model of lipoprotein lipase. Polar contacts (yellow dash line) between His154 and Arg180 lost and new interactions between His154 and Tyr315 appeared after H154R alteration. With A288T variant, a new repulsion formed between Thr288 and Ile232 (red cylinder). **(E)** Multiple alignments of LPL protein sequence across 13 species. Mutation sites His154 and Ala288 are labelled by a red rectangular. *in the last row indicates amino acid in this site is “highly conserved”.

WES was applied to support the diagnosis. We identified a pair of compound-heterozygous variants, c.862G>A (p.A288T) and c.461A>G (p.H154R), in *LPL* gene (NM_000237, NP_000228) and validated them by Sanger sequencing (Figure 1C). Variant c.862G>A (chr8:19813438) is a missense, which leads to an alteration of protein sequence (p.A288T) (Figure 1D). The variant is located in a well-established functional domain (N-terminal lipase) (PM1). It is absent from controls or at extremely low frequencies (ESP6500, 0; ExAC, 4.12E-05; gnomAD, 5.97E-05; 1kGenome, 2.00E-04) (PM2_supporting). Variant c.862G>A has been detected *in trans* with pathogenic mutation c.836T>G (p.L279R) (PM3) (Ma et al., 1994). Multiple lines of computational evidence support the variant will cause a deleterious effect on its products (MutationTaster2, score = 1.000, disease causing; SIFT, score = 0.001 < cutoff = 0.05, damaging; Provean, score = −3.71 < −2.5, deleterious; Polyphen-2, HDIV score = 0.986 > 0.957, HVAR score = 0.933 > 0.909, probably damaging; LRT, deleterious; FATHMM, score = −3.17 < −1.5, damaging; Mutpred2, score = 0.923 > 0.7, pathogenic, gain of phosphorylation) (PP3). Phenotypes of the patient are specific for LPLD (PP4). This variant has been recorded in dbSNP (rs1800011) and reported as likely disease-causing mutation (DM?) in multiple unrelated LPLD patients with the same phenotype according to HGMD database (PS4_moderate and PP5) (Ma et al., 1994; Rodrigues et al., 2016; Jin et al., 2018; Tada et al., 2019). Thus, c.862G>A (p.A288T) is classified as “likely pathogenic” (PS4_moderate+PM1+PM2_supporting+PM3+ PP3+PP4+PP5) according to the ACMG guidelines.

Variant c.461A>G (chr8:19810852) is also a missense, resulting in an amino acid change from histidine to arginine (p.H154R) (Figure 1D). This variant is a novel one, which has not been reported in any cases before. It is located in a functional domain, N-terminal lipase domain (PM1) and is absent from controls (gnomAD, ExAC, dbSNP, 1kGenome) (PM2_supporting). It has been detected *in trans* with likely pathogenic mutation c.862G>A (PM3). Multiple lines of *in silico* algorithms predicted the variant deleterious (MutationTaster2, score = 1.000, disease causing; SIFT, score = 0.001 < 0.05, damaging; Provean, score = −7.65 < −2.5, deleterious; Polyphen-2, HDIV score = 0.983 > 0.957, HVAR score = 0.962 > 0.909, probably damaging; LRT, deleterious; FATHMM, score = −3.36 < −1.5, damaging; Mutpred2, score = 0.875 > 0.7, pathogenic, loss of catalytic residue) (PP3). Phenotypes of the patient are specific for LPLD (PP4). His154 is highly conserved across species (Figure 1E). Therefore, novel variant c.461A>G (p.H154R) is classified as “likely pathogenic” (PM1+PM2_supporting+PM3+PP3+PP4).

### 3.5 Treatment and Prognosis

**FIGURE 2:**
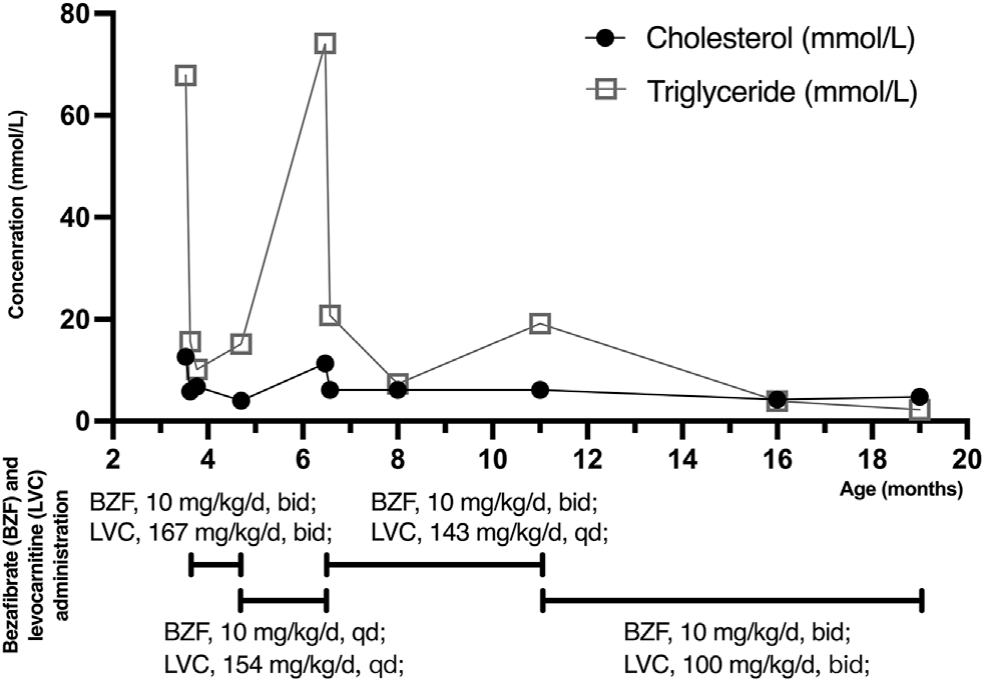
Cholesterol (CHOL) and triglyceride (TG) levels with bezafibrate (BZF) and levocarnitine (LVC) administration. The patient was admitted to hospital with high levels of CHOL (12.58 mmol/L) and TG (67.84 mmol/L) at the age of 3 months and 16 days. Three days later, her CHOL and TG levels plummeted due to bezafibrate and levocarnitine administration. The levels kept decreasing until she was discharged. Her parents reduced the dose without professional medical advice, resulting in a severe relapse (a sharp increased TG level at the age of 6 months and 14 days). The dose of medications was then adjusted and CHOL and TG levels of the patient finally declined to normal and almost normal. (TG: 1 mol/L = 88.5 mg/dl).

The patient was admitted to hospital due to fever and vomiting with extremely high levels of TG and CHOL in the blood (Table 1). Anti-infective and symptomatic treatment was applied (Figure 2). Two days later, her symptoms of fever and vomiting disappeared, with a dramatic drop of TG and CHOL levels. Then, bezafibrate (10 mg/kg/d, bid) (60 mg/d) and levocarnitine (167 mg/kg/d, bid) (1 g/d) were administrated to enhance lipoprotein lipase activity and promote the β-oxidation of fatty acids, thereby reducing TG levels. As the patient gains weight, the dose of medications changed. During the treatment, TG and CHOL levels continued to decrease towards normal. Her apoprotein levels also recovered. Her LDL level increased and HDL level declined (still out of reference values). However, after discharge, her parents reduced the dose to a half (bezafibrate, 30 mg/d; levocarnitine, 0.5 g/d) without professional medical advice, resulting in a serious relapse (a sharp rise in TG levels at the age of 6 months and 14 days). The dose of bezafibrate was then adjusted to 60 mg/d (9 mg/kg/d, bid). Four and a half months later (the age of 11 months), the dose of bezafibrate and levocarnitine was adjusted to 100 mg/d (10 mg/kg/d, bid) and 1 g/d (100 mg/kg/d, bid), respectively. The patient’s blood lipid levels finally fluctuated to nearly normal.

## 4 Discussion

**FIGURE 3:**
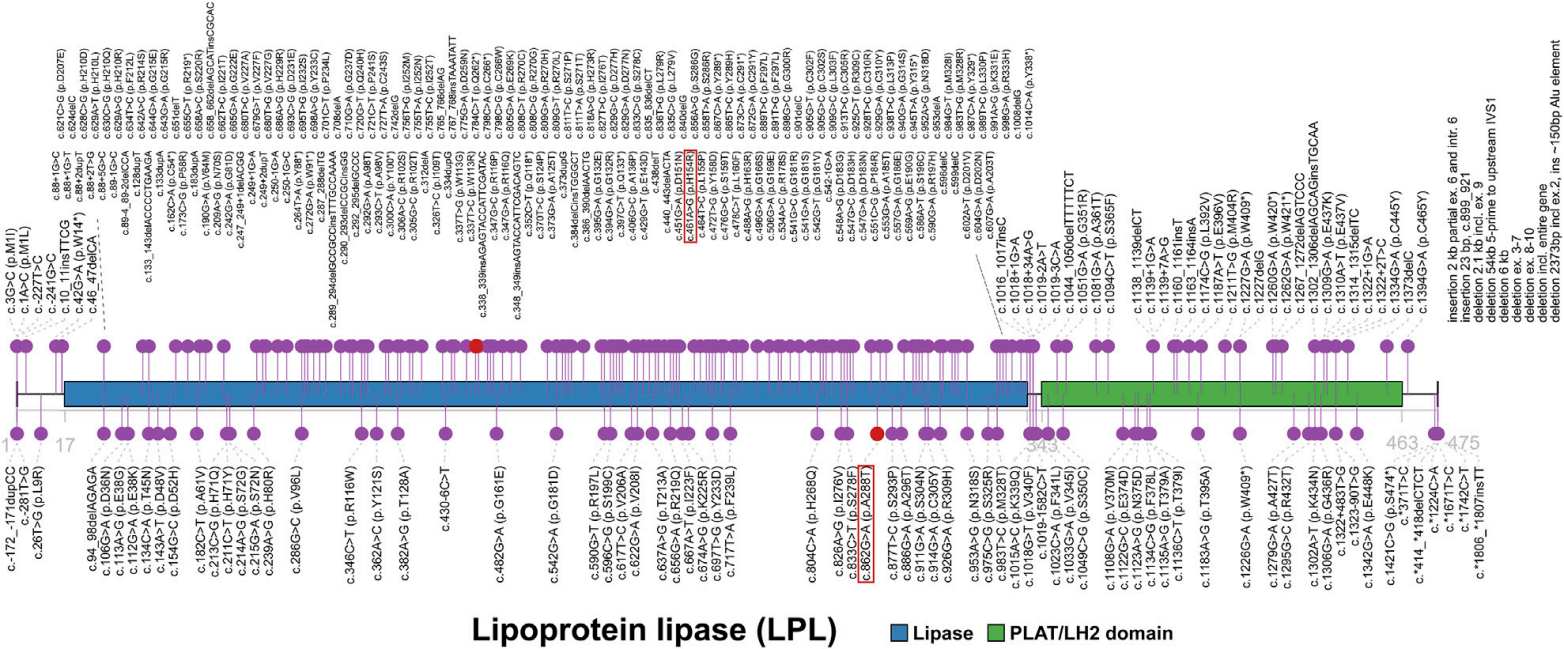
The spectrum landscape of mutations in *LPL* gene. Lipoprotein lipase (1-475) consists of lipase (17-338) and polycystin-1, lipoxygenase, alpha-toxin or lipoxygenase homology (PLAT/LH2) domain (343-463). A total of 287 mutations and polymorphisms of *LPL* gene were reported in HGMD. Disease-causing mutations (DMs) are marked in the top. Other non-DMs and polymorphisms are marked in the bottom. Two variants identified in our case are highlighted in red square.

The *LPL* gene, located on chromosome 8p21.3, encodes the lipoprotein lipase which is the key enzyme of triglyceride metabolism. LPL has both triglyceride lipase and phospholipase activities in the physiological condition. It acts mainly as a triglyceride lipase with very low phospholipase activity (McCoy et al., 2002). LPL catalyzes the hydrolysis of TGs to remove lipids from circulating TG-rich lipoproteins, specifically chylomicrons and very-low-density lipoproteins (VLDL), and thereby plays a critical role in lipid utilization and storage (Weinstock et al., 1995; Lutz et al., 2001; Pingitore et al., 2016). Lipoprotein lipase consists of two domains, lipase domain (17-338) and polycystin-1, lipoxygenase, alpha-toxin or lipoxygenase homology (PLAT/LH2) domain (343-463) (Figure 3). Same as the majority of mutations, both current variants are located on lipase domain, indicating the importance of its function and integrity. Mutations in *LPL* gene reduce or eliminate the activity of lipoprotein lipases. The absence of fully functional LPL disrupts the normal breakdown of TGs, preventing adequate clearance of circulating TG-rich lipoproteins. This impaired TG hydrolysis contributes to severely elevated TG levels (usually >16.95 mmol/L [1500 mg/dl]) in plasma (Chait and Eckel, 2019). Moreover, TGs accumulates in the blood vessels and tissues, resulting in pancreatitis, hepatosplenomegaly, eruptive xanthomas and other uncommon symptoms. Although variants in *LPL* gene usually caused lipase deficiency, the enzyme has been found overactivated in some cases, associating with lower TG levels (Smith et al., 2010). In this patient, two missense mutations resulted in reduced activity of LPL rather than complete deficiency, as bezafibrate effectively enhanced the activity of lipoprotein lipase.

There are totally 287 mutations and polymorphisms of *LPL* gene in HGMD (Figure 3), including 214 (74.6%) DM, 58 DM? 3 disease-associated polymorphisms (DP), 8 disease-associated polymorphisms with additional functional evidence (DFP) and 4 *in vitro* or *in vivo* functional polymorphisms (FP). Of these, all of 214 DMs were reported to cause LPLD or associated phenotypes clearly. In terms of the type of variants, HGMD collected 118 missense, 44 small insertion/deletion (41 frameshift and 3 inframe), 18 splice (16 canonical), 18 nonsense, 9 large insertion/deletion (>21 bp), 2 regulatory, 3 noncoding and 2 initiation. Missense mutations account for more than half (118/214, 55.1%) of all DMs and are a common mechanism of LPLD, thereby enhancing the likelihood that the two variants reported here cause disease.

Common clinical features of LPLD include recurrent abdominal pain, acute pancreatitis, hepatosplenomegaly, eruptive xanthomas and lipemia retinalis. Some of these symptoms can be fatal for the patient, but none occurred in our patient. We attribute this to the early detection. The combination of results from biochemical examinations and genetic sequencing provides a clear direction for clinical diagnosis. More importantly, it has also led to early diagnosis and proper intervention. For LPLD patients, palliative treatment and medical nutrition therapy are commonly given depending on symptoms. Blood lipid-lowering medicines such as bezafibrate are usually administrated to negatively regulate blood lipid concentrations (Bezafibrate Infarction Prevention, 2000). The maintenance of plasma TGs at less than 11.30 mmol/L (1000 mg/dl) could prevent severe complications such as severe pancreatitis, further preventing the high morbidity and mortality rate (Garg and Rustagi, 2018). In the current case, the concentrations of plasma TGs and CHOL in the patient sharply declined under the action of bezafibrate and levocarnitine. Since this early and proper administration of drugs, the patient’s lipid levels are maintained at a normal level while severe complications did not occur. Meanwhile, medium-chain TGs are also recommended for daily diet, as they could be absorbed directly into the portal vein independent on chylomicrons (Burnett et al., 1993).

In summary, this study reported a novel pair of variants in *LPL* gene causing LPLD in a 3.5-month-old Chinese girl. The novel variant, c.461A>G (p.H154R), further expended the disease-causing mutation spectrum of LPL deficiency. *LPL* mutations reported in HGMD were also reviewed and further analyzed. Missense mutations and mutations located in lipase domain account for the majority. Genetic screening can provide conclusive evidence for diagnosing LPLD. Our study and review are supportive for the early diagnosis and proper treatment of patients with LPLD in the future.

## Data Availability

The datasets presented in this study can be found in online repositories. The names of the repository/repositories and accession number(s) can be found in Sequence Read Archive (SRA) database, PRJNA761861.
