# Supplementary material for: Case Report: Next-Generation Sequencing Identified a Novel Pair of Compound-Heterozygous Mutations of LPL Gene Causing Lipoprotein Lipase Deficiency
Source: Front Genet. 2022 Mar 3;13:831133. doi: 10.3389/fgene.2022.831133 (PMC8927541; doi:10.3389/fgene.2022.831133)
Supplement: Supplementary file 1 [file DataSheet1.docx]

**Sup Table 1**. Blood lipid and lipoprotein levels in other family members.

| **Family member** | **CHOL** (mmol/L) |  | **TG** (mmol/L) |  | **HDL** (mmol/L) |  | **LDL** (mmol/L) |  | **ApoA1** (g/L) |  | **ApoB** (g/L) |  |
| --- | --- | --- | --- | --- | --- | --- | --- | --- | --- | --- | --- | --- |
| Father (III-2) | 4.64 | - | 1.18 | - | 0.76 | ↓ | 3.86 | ↑ | 0.96 | ↓ | 1.21 | - |
| Mother (III-3) | 4.42 | - | 1.03 | - | 1.79 | ↑ | 2.68 | - | 1.65 | - | 0.82 | - |
| Brother (IV-2) | 4.07 | - | 0.73 | - | 1.13 | - | 2.96 | ↑ | 1.15 | - | 0.92 | - |
| Grandpa (II-3) | 4.37 | - | 1.30 | - | 0.83 | ↓ | 3.49 | ↑ | 1.02 | ↓ | 1.16 | - |
| Grandma (II-4) | 5.07 | - | 0.69 | - | 1.79 | ↑ | 3.23 | ↑ | 1.68 | - | 0.84 | - |
| Aunt (III-1) | 4.23 | - | 0.94 | - | 1.04 | - | 3.20 | ↑ | 1.11 | - | 0.97 | - |
| **Reference** | 2.8-5.7 |  | 0.32-1.46 |  | 0.9-1.74 |  | 1.55-2.86 |  | 1.1-1.95 |  | 0.45-1.4 |  |

ApoA1, apolipoprotein A1; ApoB, apolipoprotein B; CHOL, cholesterol; HDL, high density lipoprotein; LDL, low density lipoprotein; LP (a), lipoprotein (a); TG, triglyceride; TG: 1 mol/L = 88.50 mg/dL;
